# What matters to migrant women during labor and birth: Chinese mothers’ experiences in Switzerland

**DOI:** 10.1186/s12884-024-06271-y

**Published:** 2024-01-20

**Authors:** Dingcui Cai, Paulina Villanueva, Hong Lu, Basile Zimmermann, Antje Horsch

**Affiliations:** 1 Institute of Higher Education and Research in Healthcare (IUFRS), University of Lausanne, Lausanne, 1011 Switzerland; 2 School of Nursing, Peking University, 38 Xueyuan Road, Haidian District, Beijing, 100191 China; 3 Confucius Institute, University of Geneva, Rue du Général-Dufour 24, Geneva, 1211 Switzerland; 4 Department Woman-Mother-Child, Lausanne University Hospital and University of Lausanne, Lausanne, 1011 Switzerland

**Keywords:** Childbirth experience, Intrapartum care, Chinese migrants, Mothers, Fathers, Grandparents, Qualitative, Switzerland

## Abstract

**Background:**

In Switzerland, foreigners account for 25.3% of the permanent resident population, and the fertility rate of migrant women is higher than that of Swiss women. However, migrant women from non-European countries are more likely to report having negative childbirth experiences than Swiss women. For example, during pregnancy, Chinese migrant mothers often felt dissatisfied with the follow-up pregnancy complications and lacked information on medical procedures and prenatal courses. In this paper, we explored their childbirth experiences in Swiss hospitals and how Swiss healthcare providers supported them.

**Method:**

A qualitative study employing in-depth, semi-structured interviews was conducted with 14 Chinese mothers and 13 family members. All interviews were audio-recorded, transcribed, and translated into English for data analysis. Thematic analysis was employed to generate a detailed description.

**Results:**

Three main themes were extracted from the transcripts: (1) Sense of security, (2) Intrapartum care, and (3) Postpartum needs.

**Conclusions:**

Our study shows Chinese migrant mothers prioritized giving birth in a physically and psychologically safe environment, with pain control and practical and emotional support from their intimate partners. They desired a physiological labor and birth with minimal obstetric interventions. Our research also reveals their postpartum needs, emphasizing the importance of postpartum support and obtaining culturally sensitive care during their postpartum hospital stay. The study adds new knowledge of specific migrant studies in Switzerland, as called for by the Swiss Federal Office of Public Health. The results call for the transcultural care skills training of Swiss healthcare providers to enable migrant women to have a more positive childbirth experience.

## Background

International migration^1^ is increasing globally. There were 281 million international migrants worldwide in 2020, nearly half (48%) of whom were women [1]. Migrant women are often young and of childbearing age [1]. Pregnancy is a period of increased vulnerability for migrant women since the transition to motherhood and migration to a new country both involve more profound changes in their psychological, social, and physical domains than any other developmental stage of the family life cycle [2, 3]. In Switzerland, foreigners account for 25.3% of the permanent resident population, of which more than half (56.47%) have lived for more than ten years or were born in Switzerland [4]. On average, the fertility rate of migrant women (2.9 children per migrant woman) in Switzerland is higher than that of Swiss women (1.8 children per Swiss woman) [4]. However, migrant women from non-European countries are more likely to report having a negative childbirth experience and being less satisfied with their maternity care than Swiss women [5, 6]. They were reported to have a higher risk of perinatal complications (e.g., cesarean section, preterm birth, low newborn birth weight, neonatal intensive care), mental and physical illnesses before or after birth, and higher infant and maternal mortality rates than native women [7]. The reasons leading to maternal health disparities have been identified as personal issues (e.g., a lack of social support and health literacy, uncertain residency status, poor economic status, and language and communication barriers) and structural problems (e.g., the burden of healthcare payments, a lack of information, and real or perceived discrimination [5, 8–10]). Maternal health among the migrant population is a public health issue that can result in significant mental distress, disrupt maternal bonding, and have long-term impacts on the well-being of the woman, the baby, her family, and wider society [11, 12]. Therefore, a better understanding of migrant women’s reproductive health is of great public health concern in the Swiss healthcare system [6].

Chinese migrants constitute a relatively small minority in Switzerland. The number of Chinese women giving birth is relatively small but steadily increasing, with 3902 Chinese mothers having given birth in the past ten years (2011-2021) – the triple when compared with two decades ago [13]. Pregnancy, childbirth, and the postpartum period are a psychological and physical continuum [14]. According to our first study on the pregnancy experiences of Chinese mothers in Switzerland, they felt dissatisfied with the follow-up pregnancy complications and lacked information on medical procedures and prenatal courses [15]. Thus, we emphasize the importance of follow-up research on their childbirth experiences. Furthermore, Chinese migrants constitute the third largest Asian migrant group in Switzerland [15]. Women from Asia tend to experience greater cultural and language gaps than those from European countries. We believe that some of the findings of this study may contribute to a better understanding of the childbirth experiences of mothers from Asia in Switzerland. The objectives of this study were to examine the childbirth experiences of Chinese migrant mothers and their family members (e.g., women’s partners, parents, and in-laws) and to identify their intrapartum care needs. Our study responds to the call of the Swiss Federal Office of Public Health for specific studies of migrant populations [7]. The findings of this study may help address health inequities and promote equal maternity care services for the migrant population from non-European countries in Switzerland. This study also enriches the body of knowledge about migrant studies on reproductive health globally.

## Methods

### Research design and participants

This is the second study in a series about the pregnancy, labor and birth as well as postpartum experiences of Chinese migrants in Switzerland [15]. A qualitative study employing in-depth, semi-structured interviews was conducted with Chinese mothers and their family members on their childbirth experiences in Switzerland. To be eligible for the study, Chinese mothers were required to give birth in Swiss hospitals. The newborns were less than one year old at the time of data collection. Women’s family members, including their partners, parents, or in-laws, did not have to be Chinese nationals. Participants were recruited from eight cantons (states) of French- and German-speaking Switzerland using a Chinese social media platform, named WeChat, by the first author (DC). Research flyers containing information about the study objectives, interview procedures, and eligibility criteria were posted in several WeChat groups of Chinese migrants in Switzerland. Potential participants were contacted by the first author (DC) to address their inquiries regarding interview procedures and data confidentiality. Once they agreed to participate in the study, interviews were scheduled either at home or online. Each participant provided their written consent to use audio recordings and transcriptions in data analysis, paper writing, and publication. The study was approved by the cantonal research ethics committee (project number: 2019–01734).

### Interview procedures

In-depth interviews took place between September 2019 and April 2020 with 27 participants, including 14 Chinese women and 13 family members. Individual interviews with 14 Chinese women and 10 Chinese family members were conducted in their native language (Standard Mandarin Chinese) and three foreign partners in English. Twenty-two participants were interviewed face-to-face at the participants’ homes whereas interviews with the other five participants were performed online.

Interviews with women and their family members were conducted using two separate interview guides with both broad open-ended questions and specific follow-up questions. Topics covered included choice of labor hospitals, admission to the hospital for labor, birth modes, labor pain, obstetric interventions, birth companions, breastfeeding initiation and practices, infant and maternal care, medical treatments, etc. The interview lasted an average of 41 minutes. All interviews were audio-recorded, transcribed, and translated into English for data analysis (three English interviews were transcribed directly into English).

### Data analysis

Thematic analysis was employed to generate a detailed description. Six steps of the analytic process from Braun and Clarke were followed [16], using qualitative analysis software MAXQDA [17].

The participants in the research team come from a number of disciplines. The doctoral candidate (DC) in Nursing Sciences is specialized in maternity care. A postgraduate (PV) specializing in psychology assisted with data analysis. Three senior researchers in Nursing Sciences (HL), Social Sciences (BZ), and Clinical Psychology (AH) have expertise in maternity care, perinatal mental health, qualitative research, and sinology. The data were analyzed by the two researchers (DC and PV) separately and then jointly discussed with AH. Regular discussions between the two researchers were organized to refine the codes and reconcile discrepancies. Both researchers worked during the analytic stage under the supervision of AH.

## Results

The 14 Chinese mothers, aged 34 on average, had been in Switzerland for one to twenty-one years. 13 out of 14 were married. All Chinese mothers had legal status and were covered by mandatory health insurance. Interviews were conducted within seven postpartum months on average. More information about the socio-demographic characteristics of the participants can be found in [15]. Birth-related information is summarized in Table 1. Three main themes and seven sub-themes were extracted from the transcripts: Sense of security, Intrapartum care, and Needs beyond childbirth. The outline of the main themes and sub-themes is shown in Table 2.

**Table 1:** Birth-related information of Chinese migrant mothers ($n=14$)

	Mean (SD/n)
**Birth hospitals**	
University hospital	7
State public hospital	4
Private hospital	4
**Birth mode**	
Vaginal birth	10
Obstetric interventions	
Artificial rupture of membranes	2
Oxytocin	4
Episiotomy	4
Epidural anesthesia	9
Instrument-assisted birth	
Forceps	1
Vacuum	2
Emergency cesarean section	3$^{a}$
Elective cesarean section	1
**Average days of postpartum hospitals stay**	4 (SD=0.75)
**Average postpartum months when interviewed**	7 (SD=4.02)

$^{a}$ The mothers switched to emergency cesarean sections when vaginal births were no longer viable

**Table 2:** Main themes and sub-themes of this study

Main themes	Sub-themes
**Sense of Security**	Preferences of birth hospital
	Timing of labor admission
**Intrapartum Care**	Pain management
	Birth mode
	Birth companion
**Postpartum Needs**	Professional support
	Culturally sensitive care

### Main theme one: Sense of Security

Chinese mothers in our study prioritized giving birth in a physically and psychologically safe environment. Their choice of the hospital and the timing of their admission to the hospital during labor were determined by their need to feel secure.

#### Preferences of birth hospital

Participants expressed their various perspectives on safe birth when it came to choosing a hospital for giving birth. Seven of fourteen Chinese mothers gave birth at four different Swiss university-affiliated hospitals. These mothers stated that they felt safer in a university hospital, where they emphasized the importance of having access to emergency interventions from interdisciplinary collaborations for both mothers and babies in case of unexpected events occurring during the labor process.

*“There are two public hospitals close to my home: one is a state-sponsored hospital and the other is a university hospital. I purposely chose X University Hospital because my baby’s umbilical cord was wrapped around his neck in my late pregnancy and my amniotic fluid was also low. The state hospital does not have infant intensive care units. In case of an emergency during my labor, X University Hospital has better equipment and better teams, which makes me feel safe.” (05-Mother)*

Four mothers preferred to give birth in smaller state or private hospitals. They generally appreciated the friendly inpatient environment, and the personalized care from a dedicated team. They reported that they had abundant opportunities to interact with healthcare workers during the labor process when they were in doubt or seek additional support for comfort. In addition, one mother who gave birth in a private hospital highly valued the ability to actively participate in the decision to administer epidural analgesia without the need to follow the rigid guideline based on the extent of cervix dilation.

*“We chose a small hospital on purpose. [* $\cdots$ *] When we needed something, the nurses could quickly respond, which made us feel at ease. [* $\cdots$ *] I had a struggling 13-hour long birth and my midwife kept patiently explaining and encouraging me. She helped me to overcome anxiety and stress toward the end of the birth. ” (13-Mother)*

*“I stayed at hospital X (a private hospital) and they handed us a form to be filled during prenatal check-ups to give consent for epidural administration. They explained that the consent would grant me prompt adoption of pain-relief should I need it. There was no need to wait until certain cervix dilation indications. I felt very comfortable and a sense of self-control thanks to the ability to apply pain control according to my personal comfort.” (03-mother)*

Three mothers gave birth in private hospitals because their obstetricians during their pregnancies were able to be present when they were giving birth there. The continuity of care and the trusted relationships made them feel confident and secure.

*“My private obstetrician worked there. My first baby was born there. I found him to be very reliable and trustworthy.” (03-Mother)*

#### Timing of labor admission

Except for one mother who had an elective caesarean section with a scheduled admission date, all Chinese mothers who planned a vaginal birth were asked to follow the admission criteria (e.g., ruptured membranes, regular contractions, and cervical dilation) provided by their birthing hospitals and to make their own judgments about when to request labor admission. However, they reported difficulties in determining whether they met the admission criteria and were in active labor because nine of them were first-time mothers without prior childbirth experiences. Consequently, they had to return to hospitals multiple times to confirm with midwives, which caused them great anxiety and insecurity while waiting to be admitted.

*“I felt contractions and was not sure if I was about to give birth. Therefore, I called the hospital and told them I was on the way. The midwife who met me did some examinations and informed me that I would not be giving birth immediately. She advised me to go home and wait for labor to start before coming to the hospital. She stated that it was normal for first-time mothers to make several trips to the hospital.” (02-Mother)*

One mother explained that her symptoms were atypical and did not meet the list of indicators of birth signs given by the hospital, leaving her to feel confused and stressed. She finally gave birth on the way to the hospital due to a series of misjudgments.

*“My symptoms were very different from the other mothers’. When I had my second child, all I had was back pain and a sore, tight back. I did not know that was a sign of labor. [* $\cdots$ *] I called the hospital at 3 a.m., and they told me that I did not need to come to the hospital. [* $\cdots$ *] One hour later, my water broke, and I started to have very strong contractions. My husband and I decided to go to the hospital immediately [* $\cdots$ *] My baby was born in my pants while we were waiting for the taxi downstairs. ” (11-mother)*

### Main theme two: Intrapartum Care

Chinese mothers in our study expressed a desire to achieve a physiological birth with minimal obstetric intervention and to avoid an instrumental birth or an emergency caesarean section. They generally appreciated pain control and continuity of practical and emotional support from a birth companion. Three sub-themes emerged during their labor and birth process including pain management, birth mode, and birth companion.

#### Pain management

In general, Chinese mothers reported they had access to comprehensive pain management and utilized various pain relief methods or techniques throughout their labor process. For instance, non-pharmacological analgesic methods, such as moving around, changing positions, deep breathing, and birthing balls, were introduced to them in the early stages of labor. Pharmacological analgesia, including intravenous anesthesia, inhaling nitrous oxide, and morphine injections, were also given when deemed appropriate or necessary. They stated that they could also proactively request epidural analgesia if the cervix had been dilated to two to three centimeters or if those mild pain relief methods were inadequate. In our study, all 12 mothers who tried a vaginal birth used different methods of analgesia at different stages of labor, except for one mother who did not have time to benefit from analgesia due to an emergency vaginal birth.

*“At first, they gave me a small amount of intravenous analgesia, and then I inhaled nitrous oxide, but neither worked to relieve my pain. [* $\cdots$ *] Finally, they administered an epidural.” (10-Mother)*

When asked about the impact of the epidural on their labor, Chinese mothers generally gave positive feedback. Eight mothers expressed that it not only allowed them to rest and regain strength between contractions but also made it easier for them to cooperate with birth attendants.

*“I felt like I was resurrected all of a sudden (laughs).” (03-Mother)*

However, four mothers thought that their late epidural, excessive doses, and technical problems as risk factors that led to undesirable childbirth experiences.

*“When I had the epidural, my legs were anesthetized, not my belly. I kept feeling pain for 20 minutes. [* $\cdots$ *] Then they decided to give me a higher dose of anesthesia, but they added too much. I could no longer feel the pain, but I also could not feel the contractions. I could not feel anything. [* $\cdots$ *] When the contractions came, my midwife told me to push. When she told me to push, I pushed, and I completely listened to her throughout the labor.” (09-Mother)*

To avoid this situation, Chinese mothers claimed that family members, such as in-laws, pressured them not to use epidural analgesia.

*“My mother-in-law, who was in China, talked with my husband on the phone. She insisted a lot that I could not use epidural anesthesia. However, we decided to use it after my husband repeatedly confirmed its safety with the doctor.” (07-Mother)*

#### Birth mode

Except for one mother who had an elective caesarean section due to advanced maternal age (46 years), the other mothers stated that their birth attendants and obstetricians had always encouraged them to have a vaginal birth. They experienced various obstetric interventions, such as artificial rupture of membranes, numerous vaginal examinations, or prolonged use of oxytocin, during the vaginal birth attempts.

*“I was examined vaginally by interns more than 20 times during the first night. Then I had a high fever, and my baby’s heart rate became erratic. They performed an emergency caesarean section, which made me very unhappy.” (07-Mother)*

Among those mothers who attempted vaginal births, three underwent instrumental births, and three had emergency caesarean sections following a prolonged and unsuccessful vaginal birth. Four of them had a traumatic birth experience and were suffering from post-traumatic stress reactions. In addition, they developed long-term health issues, such as postpartum anemia, prolonged body pain, urine leakage, and constipation, following childbirth.

*“I was given intravenous oxytocin for three days at the hospital, and I bled vaginally on the third night. They rushed me to the delivery room and resumed the oxytocin injections. Then I suffered 20 hours of painful contractions. I requested an epidural when my cervix dilated to two fingers. Soon after, I began having nosebleeds. The doctor rushed me to an emergency caesarean. [* $\cdots$ *] I suffered too much pain from vaginal birth to an emergency caesarean section. [* $\cdots$ *] It had long-term consequences for my physical and mental health.” (05-Mother)*

Nine of the fourteen mothers considered their childbirth experiences as poor. The main reasons for their negative experiences were birth complications associated with instrumental births and emergency interventions, which did not match their expectations based on their smooth pregnancy process or their ideology about which birth mode had better childbirth outcomes. Mothers who had instrumental births felt that they should have gone straight to caesarean sections to avoid the prolonged struggles and traumatic experiences during the vaginal birth process, as well as postpartum suffering. Mothers who had emergency caesarean sections expressed dissatisfaction with the sudden change in the birth mode because they believed that such an emergency intervention was only necessary for those mothers with complications during the pregnancy, which they did not have.

*“My childbirth experience was far from what I expected. I always thought I would have a vaginal birth because my baby and I were very healthy throughout my pregnancy. I knew mothers like me who all had vaginal births. This was so sudden for me.”(04-Mother)*

*“To insert the forceps, they performed an episiotomy. I lost almost 550 ml of blood.” (09-Mother)*

Some mothers and their family members felt there was a lack of transparency in information exchange and the decision-making process when it came to finding the optimum birth mode appropriate for the mothers according to their personal circumstances and precipitations.

*“What I very much regret is that they were not transparent enough about what was happening. They performed an episiotomy on my wife, and I had no idea until I saw what was going on. [* $\cdots$ *] They did not explain to me anything from start to end. I did not think it was a good practice, anyway.” (09-Father)*

*“The nurses told me that the doctor would come the next day to discuss with us the possibility of a caesarean section. However, the next morning, the doctor informed me I would be having a caesarean section right away. I thought, ‘What? There was no discussion. It was an already-made decision.’ [* $\cdots$ *] I was very disappointed, but as minority migrants, we did not speak the language fluent enough to argue with them.” (07-Mother)*

Some mothers were hesitant to give birth vaginally due to various concerns, such as a previous caesarean section, a large fetus, or severe vaginal bleeding. They considered that a better birth outcome might be achieved if they were allowed to have an elective caesarean section (which women have the freedom to choose in their home country, China).

*“Compared to my previous childbirth experience with an elective caesarean section, I suffered more and had a lengthy recovery this time due to an instrumental birth. I think the caesarean section would have been better for me.” (09-Mother)*

#### Birth companion

In our study, all mothers, regardless of vaginal birth or caesarean section, had their intimate partners as birth companions at various stages of the labor process. Ten Chinese mothers who gave birth vaginally stated that practical support, such as interpreter assistance, non-pharmacological pain relief, and emotional support, were crucial to making them feel secure and relaxed.

*“My husband was extremely helpful, especially when I was in pain. I took prenatal classes to learn how to breathe to relieve pain, but I forgot how to do it when I was in actual labor. My husband kept encouraging me by massaging, holding my hands, and cheering me. The presence of my loved one put me at ease.” (01-Mother)*

Four Chinese mothers who had caesarean sections reported that their partners were permitted to accompany them during certain stages of the caesarean section, such as after anesthesia was administered, the operating table was prepared, or the caesarean section was performed. They wished for flexibility regarding when their partners could be present during the caesarean section. Granting earlier companionship from their partners was considered beneficial.

*“It was too late for my husband to come in! (Rising intonation). [* $\cdots$ *] He was not allowed in until after my belly had been opened. If he had been there earlier, I would not have had to keep an eye on the medical personnel around me, and I could have relaxed.” (06-Mother)*

Generally, fathers of Chinese or non-Chinese nationals all confirmed that being present during the birth process had a positive physical and psychological impact on mothers. Although some Chinese fathers described the labor and birth process as “brutal” and “unpleasant”, they believed that witnessing the birth was a highly meaningful and emotional event.

*“I found being a birth companion a special and meaningful experience for me. The labor process was relatively long and exhausting because it was our first child. [* $\cdots$ *] At first, I thought I would not be too excited to see the baby, but after I was with my wife for more than 10 hours, I cried when I saw the baby come out. I cannot imagine how anxious I would have been if I had just waited outside the delivery room. But after I went through the entire process, all I felt was moved and heartwarming. I felt that as a father, it was very precious to witness the first moments of my child coming into this world.” (10-Father)*

### Main theme three: Postpartum needs

Chinese mothers and their families in our study desired professional support and culturally sensitive care. They expressed concerns about support for infant care and breastfeeding initiation, in particular those with caesarean sections. In addition, they desired to retain some traditional cultural practices that were still meaningful to them following childbirth.

#### Professional support

Although standardized neonatal care was practiced following childbirth, several mothers reported shortfalls in the guidance and support they received in establishing early mother-infant bonding. Mothers with cesarean section or assisted virginal birth reported issues ranging from inadequate skin-to-skin contact, and lack of mother-infant rooming-in, to weakness in practicing early bonding after excessive blood loss. They considered instructions given by maternity care workers insufficient and they often did not know what to expect after the newborn were out.

*“My son was delivered by cesarean section. They didn’t explain much about the process. I had to ask my husband whether the baby was out. When the baby was out, they brought the baby to me but only rubbed him against my face a few times before taking him away with my husband. I didn’t even have time to look at my baby.” (07-Mother)*

Early sucking and breastfeeding initiation were perceived to be very challenging for most mothers immediately following childbirth and during their hospital stay. Various difficulties were raised by delayed milk production, inverted nipples, poor infant-mother attachment, or inappropriate breastfeeding positions. Complications for breastfeeding developed in one case due to earlier formula feeding administered by maternity care works with bottle nipple, leading to baby’s nipple confusion.

*“I had no milk at all in the first three days after giving birth, even with breast pumping. My baby’s weight dropped significantly. He didn’t get anything, even though he kept sucking. The midwives suggested I turn to formula milk. They gave him formula milk in a bottle. He drank very fast. He didn’t want to suck for breast milk anymore after that. Because it was much easier for him to suck out milk with bottle nipples.” (09-mother)*

Support was deemed even more imperative by mothers following caesarean-section, whose mobilities were greatly restricted due to post-operative pains.

*“My baby started breastfeeding on the third day, but he could not suck on my nipples at all because I have inverted nipples. In addition, I had a large caesarean section wound and no idea how to breastfeed. [* $\cdots$ *] The midwives told me, ‘I will only teach you once, and then you should do it on your own,’ so I was hesitant to seek their help again.” (05-mother)*

*“They told me to go upstairs to the inpatient unit for some medical examinations after the caesarean section. This was extremely difficult for me because I was extremely weak. The wound was extremely painful, every step I took hurt terribly. [* $\cdots$ *] I thought their service was poor, and I was particularly dissatisfied.” (04-Mother)*

Unlike in China, where family members are encouraged to stay throughout the day in the hospital to assist with the care of newborns and mothers, Chinese mothers realized that Swiss hospitals do not allow family members to stay overnight. Consequently, some mothers reported that their postpartum hospital stays were very challenging, particularly for those who underwent a caesarean section.

*“The nights were the most difficult for me because I did not have anyone to help with baby care. He cried all the time while he was in the stroller. I could not even move toward the bed after the caesarean section, let alone hold and comfort him.” (05-mother)*

A few Chinese mothers and their family members believed that their healthcare providers were occasionally critical rather than helpful.

*“In general, the midwives would not help us with infant care. [* $\cdots$ *] When the baby kept crying, the midwife came and asked, ‘Why have you not calmed him down?’ ” (08-Father)*

#### Culturally sensitive care

Chinese mothers and their family members reported that their needs for culturally sensitive care were primarily connected with their traditional postpartum practices. They claimed that it was not always possible to strictly follow their cultural customs regarding hygiene and cleanliness restrictions, as well as dietary and drinking habits. Participants explained that their healthcare providers were not aware of their specific cultural customs and thus could not provide culturally sensitive care. In addition, the lack of effective communication between mothers and their healthcare providers sometimes resulted in misunderstandings or confusion for both parties. Nevertheless, with proper guidance, Chinese mothers managed to adapt to the norms of the host culture while maintaining some cultural practices that were still meaningful to them.

*“My midwife advised me to take a shower the day after the caesarean section. I told her that in our culture, mothers are usually not allowed to shower for the first month after giving birth. The midwife warned me that if I did not shower, I would stink and bother the other mothers in the room. Eventually, I took a shower upon the midwife’s request.” (07-Mother)*

*“Chinese women are supposed to keep warm and eat hot food after giving birth. However, the hospital here was unconcerned. They served cold drinks and food to my daughter (the baby’s mother). We brought hot water and food from home to the hospital every day.” (06-Grandfather)*

## Discussion

Like most women worldwide [14, 18], Chinese mothers who gave birth in Switzerland desired a physically and psychologically safe environment. Compared to Swiss mothers, who prioritized professional and obstetric skills, an intimate atmosphere, and continuity of care from a private obstetrician when choosing a birthing hospital [19–21], the safety of both mothers and babies during the labor process was weighed heavily among Chinese migrant mothers. University hospitals were their first choices as they associated their safety with the availability of interdisciplinary collaborations in general hospitals to provide emergency interventions for both mothers and babies in case unexpected events occurred during labor and birth. A few mothers, especially from densely populated major Chinese cities, placed a higher value on woman-centered care [22, 23] from the entire maternity care team during the labor process in smaller-scale state hospitals or private hospitals. They appreciated the maternity care that prioritizes pregnant women’s caring needs, personalized aspirations for comfort, and the ability to collaborate with maternity care professionals for decision-making. All unlikely to be accessible for the general population in their home country due to “assembly-line birth” [24] in Chinese hospitals.

Evidence-based recommendations for late labor admissions can avoid early obstetric interventions, emergency caesarean sections, and instrumental births [25, 26]. However, unlike other migrant mothers who delayed hospital admission for fear of caesarean sections [27], Chinese mothers in our study perceived early admission as a safe guarantee for handling labor uncertainties during the early stage, compromising the increased probability of early and frequent medical interventions. It is tied to the strong beliefs in Chinese culture that childbirth is a very dangerous event and that a woman who gives birth to a child is passing through a ghostly gate [28]. It is important to ensure the safety of women and babies so that the event takes place without danger. Furthermore, our study also showed that the criteria for labor admission based on active birth symptoms as instructed by healthcare personnel were considered ambiguous and that one mother even experienced atypical labor symptoms. All of these posed challenges for Chinese mothers to correctly interpret their early labor symptoms and left them stressed about the proper timing to request admission. In view of supporting mothers and improving the efficacy of maternity care services, it is advisable to encourage healthcare providers to provide more practical and clear guidelines and admission procedures to migrant women. It is imperative to educate them to truly understand that the early labor process can be lengthy. Moreover, it would be beneficial to provide migrant mothers with timely phone support to facilitate the recognition of early labor reactions at home.

In contrast to previous studies of Chinese mothers giving birth in some foreign countries [29, 30], which found that Chinese mothers perceived labor pain as inevitable and tolerated it silently, almost all Chinese mothers in our study highly valued labor analgesia for pain control. The reason for such a disparity in Chinese migrant mothers’ practices could be partly attributed that epidural analgesia is widely regarded as an effective and safe for labor pain relief in developed countries [31–34]. In the meanwhile, with the evolution of childbirth ideology among Chinese people over the past decades as China underwent rapid social and economic growth and reformation, China has been advocating the implementation of epidural labor since 2015 to reduce the caesarean section rate, as fear of labor pain is the primary reason for Chinese mothers to opt for caesarean sections [35, 36]. This national campaign has also dispelled the widespread beliefs among Chinese women and their families that epidural analgesia is harmful to the mother and the baby. Consequently, Chinese mothers, as shown in our study, became more self-aware of their physical and psychological comforts rather than silently tolerating and suppressing their pain relief needs. It should also be noted that a few Chinese mothers in our study had less desirable pain control outcomes, and they attributed this to inappropriate timing of pain management at certain stages, excessively administered pain-relief medicine doses, or technical problems. This necessitates further research to understand any underlying deficiencies in the assessment of proper obstetric interventions or ineffective communications between mothers and healthcare providers.

Our research found that Chinese migrant mothers (one-third) were much more likely than Swiss women (one-sixth) to have instrumental vaginal births [37]. Nine out of ten mothers who gave birth vaginally in our study were subjected to various obstetric interventions (e.g., artificial rupture of membranes, numerous vaginal inspections, early and prolonged use of oxytocin, and lateral episiotomy) during their early labor stage that are not recommended by WHO intrapartum care guidelines [18]. Three Chinese mothers switched to a caesarean section after a long and unsuccessful trial of vaginal birth. These interventions probably played a role in an abnormal fetal heart rate, maternal fatigue, or fever, necessitating an instrumental birth or an emergency caesarean section. To avoid the traumatic childbirth experiences reported by Chinese mothers in our study, in particular for difficult instrumental births, it may have been preferable to proceed directly to a caesarean section [38]. It should also be noted that traumatic and instrumental births had been reported as a common issue in the Swiss maternity care system, not only experienced by Chinese migrant women but also Swiss maternal women in general [39, 40]. However, migrant women are more likely to confront such an issue due to communication difficulties and poor maternity care support [7].

The previous research on Chinese migrant mothers in the United Kingdom and Canada, conducted nearly 20 years ago, showed that mothers hesitated to have their partners present during childbirth to avoid embarrassment out of fear of their loss of control and emotional outbursts over labor pain [29, 41]. However, Chinese mothers in our study highly appreciated the birth companionship, which provided them with a great sense of security and confidence. They placed a high value on psychological safety and received practical and emotional support from their birth companions. This is particularly important for migrant mothers in an unfamiliar healthcare environment. This should be viewed in the context of the unique setting for Chinese mothers giving birth in Switzerland. Unlike in English-speaking countries, where Chinese mothers could initiate more in-depth communication with health professionals in English, a widely taught second language in China, language barriers faced by those Chinese mothers in Switzerland impede their communication with their healthcare providers [15]. Partners of those Chinese mothers who either speak the official Swiss language or have relatively adequate language skills could greatly aid communication between birth attendants and mothers. Another factor contributing to the positive experiences from partner engagement in the childbirth process is Chinese mothers’ ability to maintain self-control while cooperating with their birth companions and birth attendants in our study, thanks to epidural analgesia.

It is noteworthy that our study indicated that overall childbirth experiences of Chinese mothers in Switzerland were perceived as less satisfactory compared to their overwhelmingly positive pregnancy experiences [15]. Arguably, we should not directly compare the mothers’ experiences at different stages of childbearing. However, this disparity indeed implies a significantly higher demand for effective communication during labor and birth. Unlike consultations with healthcare providers in hospitals or clinics during pregnancy, which were carried out and repeated over an extended period, mothers need to accurately convey their rapidly changing personal situation to birth attendants during a relatively short but intense childbirth process. Given that direct and individualized communication with healthcare providers throughout the labor and birth process is essential for promptly addressing mothers’ needs or adjusting any treatment or intervention plan, strategies to overcome communication barriers between migrant mothers and healthcare providers need to be implemented. Healthcare professionals and social service providers should closely collaborate to ensure migrant populations have equal access to quality care. For example, childbirth preparation courses should be expanded to all migrant women in Switzerland, ranging from essential knowledge about pregnancy, childbirth, and breastfeeding to a focus on the maternity care system and available support resources, with the assistance of community interpreter services [42].

Previous research on migrant women’s maternity care services in Switzerland has often overlooked their postpartum experiences during the hospital stay, but mainly focused on barriers to access maternity care during the pregnancy period, antenatal services, and pregnancy outcomes [5, 9, 43, 44]. Our study investigated the experiences of Chinese mothers during postpartum hospitalization, with a particular focus on their need for support from their healthcare providers. Chinese mothers frequently expressed concerns about inadequate support for infant care and breastfeeding initiation, as well as a lack of support and attention to mothers who had caesarean sections. A few mothers and fathers felt their healthcare providers were sometimes critical rather than helpful. Given that postpartum care and support during hospitalization are primarily provided by family members in China, our study reveals a significant gap in understanding Chinese mothers’ needs and expectations between themselves and their healthcare providers. Since migrant women and their families frequently lack knowledge of the routines, practices, and personnel of the Swiss maternity care system, assisting them in understanding the coverage of postpartum support and services in the Swiss healthcare system before labor admission should be promoted. This will enable them to be prepared even in the absence of family support and to explore strategies for coping with the challenges that come with childbirth during their postpartum hospital stay.

Finally, it is interesting to observe how Chinese mothers and their family members attempted to adapt to the local practices of the host culture while retaining some traditional cultural practices that were still meaningful and accessible to them. For instance, they demonstrated flexibility in breaking hygiene and cleanliness restrictions imposed by their traditional postpartum customs while adhering to the Yin (cold) -Yang (hot) concept in Chinese traditional medicine by eating and drinking warm [45]. Healthcare workers should encourage migrant women to express their concerns and specific needs related to their cultural practices.

## Strengths and limitations

The strengths of our study include the diverse perspectives of Chinese mothers, their partners, parents, and in-laws. The interviews were conducted within seven months following childbirth on average when the mothers and their family members had fresh memories of their previous birth experiences. The findings of this study apply directly to healthy migrant mothers of various parities and economic and educational backgrounds. However, migrant women with specific conditions, such as severe prenatal comorbidities or postpartum complications, or who had a newborn baby with health issues, were not included in this study. Migrant mothers falling into this group may have different concerns and expectations when it comes to their maternity care needs. Future studies are required to build a comprehensive understanding of the maternity care needs of migrant populations in Switzerland. In our study, almost all mother participants have a high educational background and above-average socioeconomic status due to the fact that immigration for non-EU citizens to Switzerland is limited to higher education, skilled work, or cross-cultural marriage. Care should be taken when applying the knowledge and experiences gained in this study to other migrant groups where a broader socioeconomic status could exist.

## Conclusions

To our knowledge, this is the first qualitative study on the childbirth experiences of Chinese mothers, a small but steadily growing minority group, in Switzerland.

In general, our study showed that the childbirth experiences of Chinese mothers in Switzerland were less satisfactory compared with their pregnancy experiences. Language barriers and cultural differences hinder the establishment of a trusted relationship and effective communication between the mothers and their healthcare providers. Our study provides pivotal information for further follow-up studies to address migrant mothers’ needs and expectations and minimize the use of invasive medical interventions, which could potentially increase the risks of traumatic birth experiences.

To achieve optimal childbirth outcomes while maximizing the efficiency of medical resource utilization within the healthcare system, it is imperative for healthcare providers and policymakers to realize Chinese mothers’ perspectives on their sense of labor security, as revealed by our study to be unique compared with native Swiss mothers. They highly valued labor pain control and birth companionship, both in contrast with previous studies on the same minority group in other foreign countries. This should be viewed in the context of rapid social-economic development in their home country in the past decades, which resulted in strengthened self-esteem and self-awareness of their own physical and psychological comfort during labor and birth.

Postpartum care for migrant mothers and their babies during their hospital stay was overlooked in previous studies. Our research followed up with Chinese mothers and indicated inadequate support for them during postpartum hospitalization to initiate breastfeeding or formula feeding. Chinese mothers, in particular those with caesarean sections, experienced difficulties caring for the babies and themselves in the absence of family members and an unfamiliar healthcare system.

When caring for migrant populations, healthcare providers need to be aware and knowledgeable of their specific cultural practices relating to pregnancy and childbirth in order to provide safe and respectful care. Further research is recommended from the perspective of maternity care providers to investigate their experiences and perceptions of caring for migrant women or those other minority groups within the Swiss maternity system, particularly those of non-European origins with very different cultural backgrounds.

## Data Availability

The individual data set created during the current study is not accessible to the general public due to concerns about potential privacy violations involving the participants. The corresponding author, however, is willing to provide the final data set for data analysis upon reasonable request.

## Footnotes

1 The movement of persons away from their place of usual residence and across an international border to a country of which they are not nationals [1]
